# Tips for writing a case report for the novice author

**DOI:** 10.1002/jmrs.18

**Published:** 2013-07-21

**Authors:** Zhonghua Sun

**Affiliations:** Discipline of Medical Imaging, Department of Imaging and Applied Physics, Curtin UniversityPerth, Western Australia, Australia

**Keywords:** Case reports, publishing, research, writing

## Abstract

A case report is a description of important scientific observations that are missed or undetectable in clinical trials. This includes a rare or unusual clinical condition, a previously unreported or unrecognized disease, unusual side effects to therapy or response to treatment, and unique use of imaging modalities or diagnostic tests to assist diagnosis of a disease. Generally, a case report should be short and focussed, with its main components being the abstract, introduction, case description, and discussion. This article discusses the essential components of a case report, with the aim of providing guidelines and tips to novice authors to improve their writing skills.

## Introduction

For many doctors and other healthcare professionals, writing a case report represents the first effort at getting articles published in medical journals and it is considered a useful exercise in learning how to write scientifically due to similarity of the basic methodology.^1^ Case reports aim to convey a clinical message.^2^,^3^ Despite different types of case reports, they all aim to enhance the reader's knowledge on the clinical manifestations, the diagnostic approach (with a focus on imaging modalities for case reports published in medical imaging/radiology journals), or the therapeutic alternatives of a disease.^2^–^4^ Thus, a case report worthy of reading should contain both useful practical messages and educational purpose.^2^–^5^

Although case reports are regarded by some as the lowest (some even do not list the case reports at all) in the hierarchy of evidence in the medical literature, publishing case reports allow for anecdotal sharing of individual experiences, providing essential sources of information for the optimum care of patients. In the hierarchy of evidence-based medicine, randomized controlled trials are placed at the top, superseded by systematic reviews and meta-analyses, followed by prospective experimental trials, then observational studies, case–control studies, and case series at the bottom.^1^,^6^–^8^ Most authors are now aware of the impact factor of journals to which they submit their studies. Case reports are infrequently cited, and therefore, publishing case reports is likely to decrease the journal's impact factor.^9^ This has led many editors to remove case report sections from their journals.^10^

On the other hand, it has been pointed out by others that case reports that are carefully prepared and interpreted with appropriate caution play a valuable role in both the advancement of medical knowledge and the pursuit of education.^11^–^16^ Vandenbroucke^17^ listed five roles of potential contribution to defend the publication of case reports:

1. Recognition and description of a new disease
2. Recognition of rare manifestations of a known disease
3. Elucidation of the mechanisms of a disease
4. Detection of adverse or beneficial side effects of drugs (and other treatments)
5. Medical education and audit

**Figure 1:**
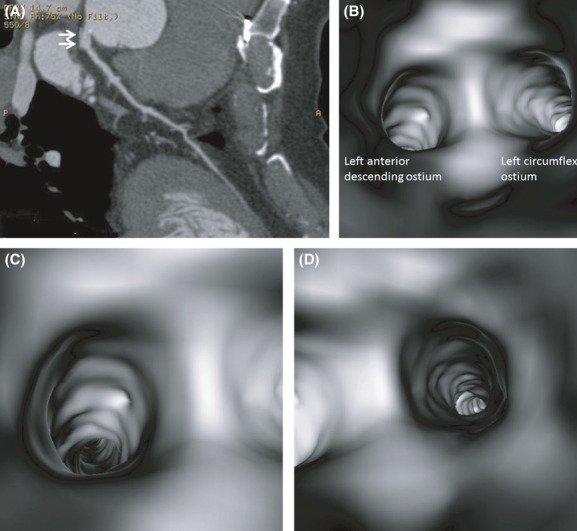
Multiplanar reformatted image showing the left coronary artery with coronary stent implanted (arrows) at the ostium of left main stem (A). Virtual endoscopy views of the proximal segment of left coronary artery (B), left anterior descending (C), and left circumflex (D). The internal wall of these coronary branches looks smooth on virtual endoscopy images with no sign of intraluminal irregularity. (Reprint with permission from Reference.^18^)

Two main roles are recognized for case reports published in medical imaging and radiology journals: as sources of new knowledge and as important means for education and learning. The case report as a source of new knowledge refers to visualization of a new manifestation or finding, or clearer demonstration of a known feature of a disease, using a new imaging technology or an imaging method.^18^,^19^ Figure 1 is an example showing 3D virtual endoscopy and the unique intraluminal views of the coronary lumen provided by this new visualization tool.^18^ The case report as a means for teaching and learning can be manifested as publication of characteristic and instructive cases for educational features. An example is that *British Journal of Radiology* (BJR) used to publish six to seven case reports in its monthly issue; however, it has changed the format to publishing “Case of the Month” since May 2012. Educational value instead of extreme rarity is the main virtue of a case report worthy of publication.^2^,^3^

Writing a case report can be educational for the author as well as for potential readers.^13^ Whether in the context of reporting something potentially new or presenting an instructive example of something well known, the author's first and most important task is to search and read extensively on the topic.^20^ This article aims to provide guidance on the novice author for writing case reports. Although it is recognized that these guidelines and tips for writing case reports are insufficient for making a successful author, they do help inexperienced authors to exercise and develop basic skills needed in medical writing.

## The structure of the case report

Case reports are shorter than most other types of articles. Case reports should encompass the following five sections: an abstract, an introduction with a literature review, a description of the case report, a discussion that includes a detailed explanation of the literature review, and a brief summary of the case and a conclusion.^21^,^22^ Tables, figures, graphs, and illustrations comprise the supplementary parts and will enhance the case report's flow and clarity. Unlike original articles, case reports do not follow the usual IMRAD (introduction, methods, results, and discussion) format of manuscript organization. As the format for case reports varies greatly among different journals, it is important for authors to read carefully and follow the target journal's instructions to authors.

### The title

The title is the first component of a case report that will be read by readers. Therefore, it should be concise, informative, and relevant to the subject. The ideal title should attract the reader's attention and state the focus on a particular issue, without being too cumbersome or artificial.^23^ Redundant words such as “case reports” or “review of the literature” should be omitted, and ostentatious words such as “unique case” or “first report of” should be avoided.^1^,^5^ Table 1 lists the titles of case reports that were published in BJR (*British Journal of Radiology*) and JMIRO (*Journal of Medical Imaging and Radiation Oncology*) between 2012 and 2013.

**Table 1:** A list of case reports published in BJR and JMIRO between 2012 and 2013

*British Journal of Radiology* (BJR)	*Journal of Medical Imaging and Radiation Oncology* (JMIRO)
Severe back pain and lower extremities weakness in a young male	Case of bilateral non-traumatic subperiosteal orbital haematomas
A painful forefoot mass	Spinal arachnoiditis as a consequence of aneurysm-related subarachnoid haemorrhage
An 85-year-old male with abdominal pain and previous gastric surgery	IVC filter limb penetration of the caval wall during retroperitoneal surgery/lymph node dissection
A right atrial mass – but where is it coming from?	Haemobilia – a rare presentation of intrabiliary hydatid disease
An unusual case of duodenal beaking	Pulmonary arteriovenous malformation: a rare anterior mediastinal mass
Cystic renal mass in a patient with previous Wilm's tumour	Neuroimaging findings in acute ethylene glycol poisoning
Can you diagnose this condition on plain radiography?	Inducible myocardial ischaemia diagnosed using computed tomography dipyridamole stress myocardial perfusion technique
Unsuspected cystic left upper quadrant mass	Partial anomalous pulmonary venous return in patients with pulmonary hypertension
An uncommon cause of abdominal pain following blunt abdominal trauma	Uncommon pulmonary metastasis presenting as pulmonary infarction with tumour emboli in two cases
“Primum non nocere” – first, do no harm	Musculoskeletal CPD revision: cases from the New Zealand bone and soft tissue tumour registry
An unusual incidental finding	

IVC, inferior vena cava; CPD, continuing professional development.

### The abstract

Like other types of articles, it is necessary to include a short summary that gives an overall idea about the content of the case report. The abstract is usually quite brief and generally shorter than that for other types of articles, and it typically has a word limit of 100 words or less. The abstract should be unstructured, pose the clinical question or diagnostic problem, and provide essential information which allows for easier retrieval from electronic database and helps researchers determine their levels of interest in the case report.^5^

### The introduction

The introduction should be concise and immediately attract the attention and interest of the reader. The introduction should provide background information on why the case is worth reading and publishing, and provides an explanation of the focus of the case report, for example: “We present/report a case of ….” Merit of the case report needs to be explained in light of the previous literature, thus, a focussed comprehensive literature review is required to corroborate the author's claim in this section. The author should bear in mind that a more detailed literature review belongs to the discussion, although critical evaluation of the literature is still required.^5^ For some journals, such as BJR (case of the month), there is no Introduction section and the body of the case reports starts immediately with a description of the case.

### The case description/summary

**Figure 2:**
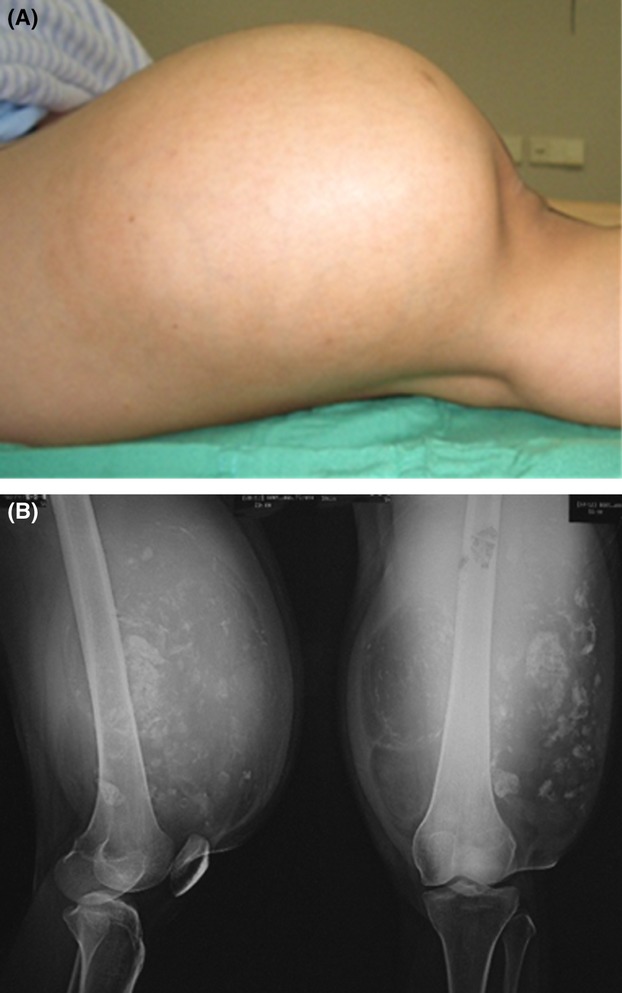
(A) Photograph showing a huge lump in the anterior part of the right thigh. (B) Radiographs revealed a bulged soft tissue mass in anterior compartment of right lower thigh showing predominantly radiolucent density with multiple chondroid matrix of calcification. Bone structure is still intact. (Reprint with permission from Reference.^25^)

The case description or summary is the focus of the case report. The case is best presented in chronological order and in enough detail for the reader to establish his or her own conclusions about the case's validity.^5^,^21^ The current medical condition and medical history, including relevant family history, should be clearly described in chronological order, typically comprising clinical history, physical examination findings, investigative results, including imaging and laboratory results, differential diagnosis, management, follow-up, and final diagnosis.^1^,^24^ The following paragraph is an example of describing the patient's history:

A 34-year-old female was admitted to the outpatient department due to an increasing lump on the right thigh, which she stated as having been present for 5 years. A painful feeling sometimes occurred in the right upper leg. There was no complaint of lower limb weakness, no history of trauma and the patient was otherwise in good health. On physical examination, a deep seated round mass was detected and located on the right thigh with a size of 25 × 25 × 15 cm, showing hard consistency and non-mobile features (Fig. 2A).^25^

All important negative findings should also be provided. The author's own interpretation or inferences should be avoided in the body of a case report. Tables/figures should be used to reveal chronological findings or to compare observations using different methods. The following paragraph is another example on the detailed description of using different methods both imaging and diagnostic:

Radiographs showed a bulge soft tissue mass in the right lower thigh having predominantly radiolucent density with multiple chondroid matrix of calcification (Fig. 2B), but the bone cortex is still intact. An MRI was obtained to further define the extent and nature of the lesion, confirming heterogeneous soft tissue mass in the anterior compartment of the muscle of the right lower thigh which mostly consisted of fat tissue, thick septation and some nodular non-adipose components. T2-weighted images through the tumour demonstrated high signal intensity comparable with the signal intensity of fat. Fat-suppressed T2-weighted images through the distal part of the tumour showed suppression of the signal through the central fatty components and lobular high signal intensity component at the peripheral rim.^25^

In particular, figures need a brief but clear description. In the case of surgery and pathology specimens, the author is advised to provide a comprehensive summary of the surgical procedure and detailed pathologist's report.^5^,^25^ The following paragraph is an excerpt from the case report published in the *Australasian Medical Journal* (AMJ):

The patient was admitted to the surgical ward with preparation for open surgery. The abdomen was opened through the site of the previous incision, and an abscess was observed and drained. A hole was detected in the peritoneal fascia. The anterior duodenum was oedematous and thickened with coverage of fibrin. A small perforated duodenal ulcer was seen. Graham patch procedure was performed to repair the perforated duodenal ulcer with two drains put in place and then the abdomen was closed. The patient was managed with intravenous fluids, as well as analgesics and antibiotics.^26^

It is worth noting that patient confidentiality must be preserved. Patient demographics such as age and gender, and occasionally, race and occupation are referred to in the first sentence. In order to reduce the possibility of identifying the patient, the patient's initials, date of birth, and other identifiers such as hospital number must not be used.

### The discussion

The discussion is the most important section of the case report. The discussion serves to summarize and interpret the key findings of the case report, to contrast the case report with what is already known in the literature and justify its uniqueness, to derive new knowledge and applicability to practice, and to draw clinically useful conclusions.^2^,^21^ In comparing the new case with prior knowledge, the author should briefly summarize the published literature and show in what aspect the present case differs from those previously published, and thus deserves to be read and published. The discussion section of a case report is not designed to provide a comprehensive literature review and citation of all references; therefore, all the references cited should be critically evaluated.

Any limitations of the case should be stated and the significance of each limitation described. The value that the case adds to the current literature should be highlighted, so should the lessons that may be learnt from the case presented, especially if new recommendations for patient diagnosis (with use of an imaging modality) or management, could be put forward.^2^,^5^,^21^ The following paragraph is an excerpt from a case report with regard to the concluding statement in the discussion:

This case report highlights the importance of using CT in making accurate diagnosis in patients with abdominal pain due to suspected GI tract perforation. In particular, appropriate selection of CT scanning protocol, such as with oral contrast administration is necessary to ensure timely diagnosis and improve patient management.^26^

In the last paragraph, the author should provide the main conclusion of the case report based on the evidence reviewed in the discussion section. A concise statement of the lesson to be learnt from the case could be stated with justifiable evidence-based recommendations. This section should be concise and not exceed one paragraph.^14^,^21^

### The references

The references listed at the end of the case report should be carefully chosen by virtue of their relevance. References should provide additional information for readers interested in more detail than can be found in the case report, and they should support any specific points highlighted.^14^ Some journals restrict the number of references to no more than 15 for a case report.

## Conclusion

A case report will not have as much potential impact on the clinical practice of healthcare as randomized controlled trials or other research articles. However, case reports provide valuable sources of new and unusual information for clinicians to share their anecdotal experiences with individual cases, make others aware of unusual presentations or complications, and deliver the educational and teaching message. Well-written and appropriately structured case reports with meticulous attention to the very minute details will contribute to the medical literature and can still enrich our knowledge in today's evidence-based medical world. Table 2 provides the suggested checklist for reporting case reports. Guidelines and tips for writing case reports are not enough for becoming a successful author; however, they are considered helpful for inexperienced or novice authors to exercise and improve their skills needed in medical writing.

**Table 2:** Checklist for writing case reports (based on advice in existing literature).^27^

Title
Should be brief and informative.
Abstract
Should facilitate retrieval with electronic searching.
Has a word limit of 100 words or less.
Introduction
Should be concise and attract the reader's attention.
Describe the uniqueness of the case and how the case contributes to the existing literature.
Is the message new and relevant to the medical imaging specialists?
Case report
Clearly describe the current medical condition and medical history in chronological order.
Provide details of the clinical presentation and examinations, including those from imaging and laboratory studies.
Describe the treatments, follow-up, and final diagnosis adequately.
Discussion
Summarize the essential features and compare the case report with the literature.
Explain the rationale for reporting the case.
State the lessons/experiences that may be learnt from the case report, and how things can be managed differently in a similar situation/case.
References
Should be relevant to the topic.
Limited to less than 15.
Figures and tables
Limited to one table, and two to three figures.
Illustrations should be effective.
